# Far-Red Light Mediated Carbohydrate Concentration Changes in Leaves of Sweet Basil, a Stachyose Translocating Plant

**DOI:** 10.3390/ijms24098378

**Published:** 2023-05-06

**Authors:** Elisa Driesen, Wouter Saeys, Maurice De Proft, Arthur Lauwers, Wim Van den Ende

**Affiliations:** 1 KU Leuven, Department of Biosystems, Willem De Croylaan 42, 3001 Leuven, Belgium; elisa.driesen@kuleuven.be (E.D.); wouter.saeys@kuleuven.be (W.S.); maurice.deproft@kuleuven.be (M.D.P.); 2 Vegobel, Missestraat 27, 2570 Duffel, Belgium; arthur.lauwers@vegobel.be; 3 KU Leuven, Laboratory of Molecular Plant Biology, Kasteelpark Arenberg 31, 3001 Leuven, Belgium

**Keywords:** far-red light, phloem unloading, polymer trapping, stachyose, starch, sucrose homeostasis, sugars

## Abstract

Photosynthetic active radiation (PAR) refers to photons between 400 and 700 nm. These photons drive photosynthesis, providing carbohydrates for plant metabolism and development. Far-red radiation (FR, 701–750 nm) is excluded in this definition because no FR is absorbed by the plant photosynthetic pigments. However, including FR in the light spectrum provides substantial benefits for biomass production and resource-use efficiency. We investigated the effects of continuous FR addition and end-of-day additional FR to a broad white light spectrum (BW) on carbohydrate concentrations in the top and bottom leaves of sweet basil (*Ocimum basilicum* L.), a species that produces the raffinose family oligosaccharides raffinose and stachyose and preferentially uses the latter as transport sugar. Glucose, fructose, sucrose, raffinose, and starch concentrations increased significantly in top and bottom leaves with the addition of FR light. The increased carbohydrate pools under FR light treatments are associated with more efficient stachyose production and potentially improved phloem loading through increased sucrose homeostasis in intermediary cells. The combination of a high biomass yield, increased resource-use efficiency, and increased carbohydrate concentration in leaves in response to the addition of FR light offers opportunities for commercial plant production in controlled growth environments.

## 1. Introduction

### 1.1. Sweet Basil: The Study Object

Sweet basil (*Ocimum basilicum* L.) is becoming an increasingly popular herb, used as a spice or for pesto production. It contains many health-promoting compounds that show anti-inflammatory, immunomodulatory, and antioxidant effects [1]. The plant is also particularly used to treat respiratory disorders [2]. Commercial breeders seek opportunities to increase yield by the stimulation of plant growth, driven by improved endogenous sugar levels.

### 1.2. Sugars in the Source/Sink Context

Sugars perform multiple tasks in plants as energy and transport molecules, carbon sources, and signaling entities [3,4,5]. Plants possess both ‘source’ and ‘sink’ tissues, with tissues producing more carbon than they consume, acting as a carbon source, and tissues using more carbon than they produce, acting as a carbon sink. Sink tissues can be completely heterotrophic plant organs (roots, seeds) or photosynthetic tissues that are not fully autotrophic [6,7,8]. To improve plant yield by means of carbon fixation for plant growth and development, the source capacity can be increased by enhancing carbohydrate production in the leaves. Additionally, the sink strength can be increased by improving the use of photoassimilates at the level of the sink tissues [7].

A small portion of the assimilated sugars produced during photosynthesis in source leaves during the light period is used for local maintenance. However, most are exported from the leaves through the phloem and allocated to sink tissues to drive their growth and development. Some sugars are polymerized into starch and temporarily stored in chloroplasts [9,10]. Starch granules consist of α-1,4-linked and α-1,6-linked glucose (Glc) polymers in the form of amylose and amylopectin [6,11], with amylopectin being the major component, contributing about 70–80% [6]. During the dark period, this starch pool is typically remobilized and transformed into sucrose (Suc) to sustain nocturnal leaf export [6,7,12,13]. In general, the monosaccharides Glc and fructose (Fru) and the disaccharides Suc and maltose are the most abundant free sugars found in plants. Hexoses mainly originate from the activity of invertases on Suc, and maltose is a typical degradation product from starch subjected to amylase activity [4].

### 1.3. Sugar Metabolism and Signaling

To ensure a relatively steady quantity of carbon to support respiration and growth under rapidly fluctuating environmental conditions, plants have evolved intricate mechanisms for carbon sensing, storage, and transport [6]. In particular, carbohydrate partitioning between source and sink tissues plays a crucial role in plant development and the yield of harvestable parts in terms of food and feed production [14]. Sugar metabolism is associated with sugar signaling. Focusing on plant growth, Glc produced by cell wall, vacuolar, or neutral invertases stimulates the TOR (target of rapamycin) signaling associated with auxin synthesis, transport, and signaling [15,16,17]. SnRK1 (sucrose-non-fermenting1 (SNF1)-related kinase1) can inhibit TOR under sugar starvation [18,19]. The SnRK1/TOR interplay is closely associated with cellular sugar homeostasis, especially Suc homeostasis and Suc/hexose ratios. These phenomena are critical to ensure normal plant development and maximize plant growth under ideal growth conditions [20], but also to control the trade-off between growth and defense under (a)biotic stress [21]. It is known that plant vacuolar invertases (VI) are closely interlinked with such sugar signaling processes [22]. Above a certain Suc threshold, VI produces the small fructan 1-kestotriose (1-K), yet another candidate signaling molecule in plants [23,24].

### 1.4. Stachyose Synthesis and Transport in Sweet Basil

Suc homeostasis is also especially crucial in the cells that are involved in phloem loading. A few plant families, such as *Cucurbitaceae* and *Lamiaceae*, to which sweet basil belongs, do not use Suc as their main transport sugar [4]. Instead, they extend Suc with one or two galactosyl units to create the raffinose family oligosaccharides (RFOs), raffinose (Raf; Gal-Glc-Fru) and stachyose (Sta; Gal-Gal-Glc-Fru), involving the cytosolic enzymes that produce galactinol (Gol), Raf, and Sta, galactinol synthase (GolS), raffinose synthase (RafS), and stachyose synthase (StaS). These enzymes function in the cytosol of intermediary cells (ICs) associated with the phloem loading of mainly Sta [25]. While the size of the IC plasmodesmata allows Suc to diffuse from mesophyll cells into the ICs, Raf and Sta are too big to flow back into the mesophyll cells. Hence, they are trapped because of their size. Thus, through “polymer trapping”, turgor pressure can build up, driving the long-distance transport of sugars [26]. In sweet basil as well as in the well-studied plant *Coleus blumei* from the *Lamiaceae* family*,* Sta is the most important transport sugar [27,28], but starch remains the most important reserve carbohydrate [28,29]. Although the symplastic way of phloem loading is energetically favorable, maintaining Suc homeostasis in ICs is crucial, since too low Suc leads RafS to act as a Gol hydrolase [30], potentially arresting RFO synthesis. Theoretically, this can be counteracted by increasing leaf starch reserves to ensure a stabilized and continuous supply of Suc for RFO synthesis. Thus, starch and Suc are not only key components for carbohydrate partitioning at the whole plant level [6,9] but also at the micro level of the cells involved in phloem loading.

### 1.5. Effect of FR Light Treatments on Plant Production

Far-red (FR) light (701–750 nm) has received substantial attention in recent years because of its synergistic effect with shorter wavelengths, increasing photosynthetic rates [13,31,32]. Adding FR light to shorter wavelengths (within the PAR range 400–700 nm) increases the quantum yield of photosystem II and reduces the non-photochemical quenching of fluorescence. It is hypothesized that FR light influences the excitation of photosystem I, which consequently leads to a faster re-oxidation of the plastoquinone pool. This facilitates the re-opening of photosystem II reaction centers, resulting in the more efficient absorption of photons [31,33]. Apart from increasing photosynthesis when applied together with shorter wavelengths and consequently increasing source capacity, FR light has also been proven to increase the sink strength of tomato fruits [13].

### 1.6. Research Objectives

The use of end-of-day FR (EOD-FR) has been proposed as an alternative use of supplemental FR light. As is also the case for FR, EOD-FR treatments can evoke the shade avoidance response, leading to stem elongation, while being hypothesized to be more energy efficient compared to adding FR light during the total photoperiod [34,35,36]. However, no reports have been found comparing the effects of continuous FR (BW + FR) and EOD-FR [35]. Therefore, this study aims to investigate the effects of both light treatments and to compare these with a broad white light background (BW). On the one hand, the effects of additional FR (peak at 730 nm) on different morphological parameters, including plant height and biomass, were evaluated. On the other hand, carbohydrate concentrations in the top and bottom leaves were measured to examine how additional FR light affects leaf source strength. In our sweet basil experimental system, we considered the source strength of two layers of leaves and measured the plant height as a proxy for the sink strength of the stem, mainly growing through cellular elongation. With this research, we aim to answer the following questions, which have not been addressed in past research: (1) Does FR light affect carbohydrate concentration in the top and bottom leaves of sweet basil, and does this effect differ between the timing and longevity of added FR light (BW + FR versus BW + EOD-FR)? (2) How do leaf carbohydrate concentrations evolve during a diurnal cycle? (3) Does FR light affect carbohydrate partitioning within the plant by increasing plant height and the stem/leaf biomass ratio?

## 2. Results

The plant morphology parameters at the end of the experimental period (37 days after sowing) are summarized in Table 1. FR radiation significantly increased plant height, with an increase of 68% (BW + FR) and an increase of 49% (BW + EOD-FR). The chlorophyll level of the oldest leaves (LN1, leaf number 1) was not significantly different between treatments, while the chlorophyll level of the youngest leaves (LN3) was significantly higher with additional FR. Adding FR light also significantly increased the contribution of the stems to the fresh and dry mass, independent of the application period. Leaf-to-stem ratios significantly decreased under additional FR, both for BW + FR and BW + EOD-FR. Adding FR to the light spectrum increased the fraction of fresh and dry mass partitioning to the stems from 19% to 16% (BW), 27% to 26% (BW + FR), and from 27% to 29% (BW + EOD-FR), respectively. However, in the case of EOD-FR treatment, the increased fresh and dry mass of the stems arrived at the expense of that of the leaves, with a similar fresh and dry weight of the leaves to the control (BW) (Table 1). Contrarily, adding FR (BW + FR) also resulted in a significant increase in the fresh and dry weight of the leaves (Table 1), thus not sacrificing leaf biomass production.

**Figure 1:**
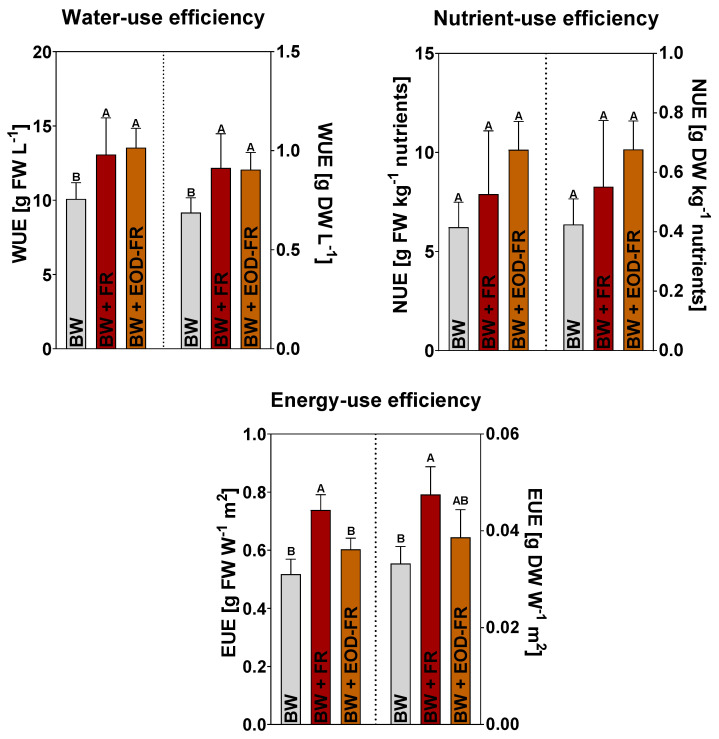
Water-use efficiency (WUE, expressed as g FW or DW L^−1^), nutrient-use efficiency (NUE, expressed as g FW or DW kg^−1^ nutrients), and energy-use efficiency (EUE, expressed as g FW or DW W^−1^ m^−2^) ± SD of sweet basil plants grown under LED light with a broad white light spectrum in the PAR range (BW), a broad white light spectrum with an additional FR peak at 730 nm (BW + FR), or under a broad white light spectrum with an additional FR peak at 730 nm during the last 2 h of the photoperiod (BW + EOD-FR). Letters indicate significant differences at *p* < 0.05 (*n* = 6) according to Tukey’s HSD test.

Adding FR to the light spectrum, either during the total photoperiod or during the last 2 h, significantly increased the resource-use efficiency. The highest values for water-use efficiency (WUE) were obtained for plants grown under additional FR radiation (BW + FR and BW + EOD-FR) regardless of the duration (Figure 1). WUE increased by 30% (BW+ FR, 13.08 g FW L^−^^1^) and 34% (BW+ EOD-FR, 13.54 g FW L^−^^1^) compared to BW (10.08 g FW L^−^^1^). Similar increases of 32% and 30% on a dry weight basis were noted for BW + FR and BW + EOD-FR, respectively. Although a higher nutrient-use efficiency (NUE) was noted when FR was applied, these differences were not significant compared to the control treatment (BW), both on a dry and fresh weight basis (Figure 1). Despite the increased electricity consumption when adding FR photons to the light spectrum, light energy was significantly better converted into biomass under additional FR treatments. Energy-use efficiency (EUE), calculated as the average FW/DW of leaves and stems per cumulated electricity consumption (W m^−^^2^), increased significantly under additional FR, with 43% (BW + FR) and 27% (BW + EOD-FR) on a fresh weight basis, and 42% (BW + FR) and 32% (BW + EOD-FR) on a dry weight basis (Figure 1).

**Figure 2:**
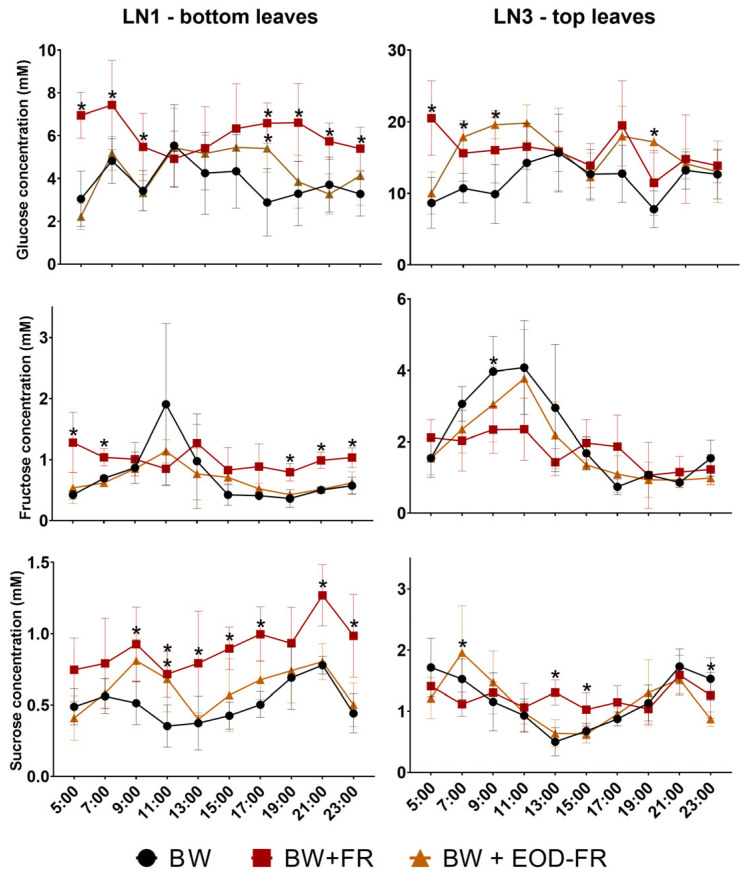
Evolution of the glucose, fructose, and sucrose concentrations (mM) ± SD during the day for the bottom (LN1) and top (LN3) leaves of sweet basil plants either grown under a broad white (BW) light spectrum, a broad white light spectrum with additional FR (BW + FR) during the total photoperiod, or a broad white light spectrum with additional FR during the last 2 h of the photoperiod (BW + EOD-FR). Significant differences against the BW control are depicted with an asterisk (*; *n* = 5, *p* < 0.05), as tested with a Tukey HSD test.

Our results revealed that FR enrichment of the light spectrum leads to the accumulation of Glc, Fru, and Suc (Figure 2). For top leaves, Glc accumulation was significantly higher at 5:00 a.m. (end of the night), 7:00 a.m., 9:00 a.m., and 7:00 p.m. for leaves of treatment BW + FR compared to BW. The results also showed that the Fru concentration in BW illuminated top leaves gradually increased in the morning, until a maximal level between 9:00 a.m. and 11:00 a.m. was reached, followed by a gradual decrease until it leveled off around 1 mM after 5:00 p.m. This gradual Fru increment and subsequent reduction in the morning was also recorded for top leaves from treatment BW + EOD-FR, but not for treatment BW + FR. The Suc concentration in the top leaves for treatment BW + FR remained remarkably stable during the day, while the Suc concentration of treatments BW and BW + EOD-FR reached a minimum at mid-day (1:00 p.m.) (Figure 2—LN3 top leaves). The FR carbon enrichment effect was more noticeable in the bottom leaves, with significant increases in the Glc concentration for the BW + FR treatment at 5:00 a.m., 7:00 a.m., 9:00 a.m., 5:00 p.m., 7:00 p.m., 9:00 p.m., and 11:00 p.m. compared to BW radiation. Similarly, the Fru and Suc concentrations of bottom leaves (LN1) from treatment BW + FR remained more stable and often significantly higher compared to the control treatment. Leaves from treatment BW + EOD-FR showed an intermediate response, but only significantly increased at 7:00 a.m. and 9:00 a.m. for Glc (top leaves), 7:00 a.m. for Suc (top leaves), 5:00 p.m. for Glc (bottom leaves), and 11:00 a.m. for Suc (bottom leaves).

**Figure 3:**
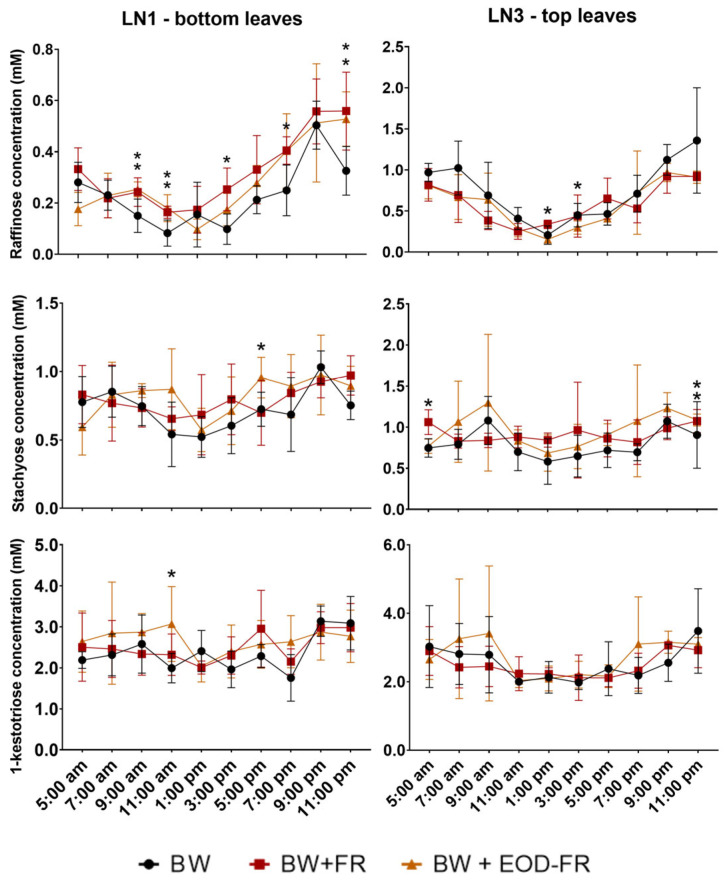
Evolution of raffinose, stachyose, and 1-kestotriose concentration (mM) ± SD during the day for the bottom (LN1) and top (LN3) leaves of sweet basil plants either grown under a broad white (BW) light spectrum, a broad white light spectrum with additional FR (BW + FR) during the total photoperiod, or a broad white light spectrum with additional FR during the last 2 h of the photoperiod (BW + EOD-FR). Significant differences against the BW control are depicted with an asterisk (*; *n* = 5, *p* < 0.05), as tested with a Tukey HSD test.

Interestingly, Raf concentrations decreased during the morning, reaching a minimum at 1:00 p.m., after which it increased again towards the beginning of the dark period (Figure 3). This pattern was not detected for Sta and 1-K, of which the concentrations remained rather constant over the measured period. Significantly higher Raf levels were detected at 1:00 p.m. and 3:00 p.m. under additional FR light. For the bottom leaves, significant differences were recorded at 9:00 a.m., 11:00 a.m., 3:00 p.m., 7:00 p.m., and 11:00 p.m. for BW + FR-treated leaves as compared to BW. BW + EOD-FR bottom leaves also showed significant increases in the Raf concentration at 9:00 a.m., 11:00 a.m., and 11:00 p.m. compared to BW. Thus, both FR and EOD-FR treatments prevented the fall in Raf levels between 10:00 p.m. and 11:00 p.m. in bottom source leaves. Strikingly, Sta concentrations for BW + FR increased significantly in the top leaves at 5:00 a.m. and 11:00 p.m., both representing measurements in the dark period. BW + EOD-FR-treated bottom leaves showed an increase at 5:00 p.m. for Sta and at 11:00 a.m. for 1-K.

**Figure 4:**
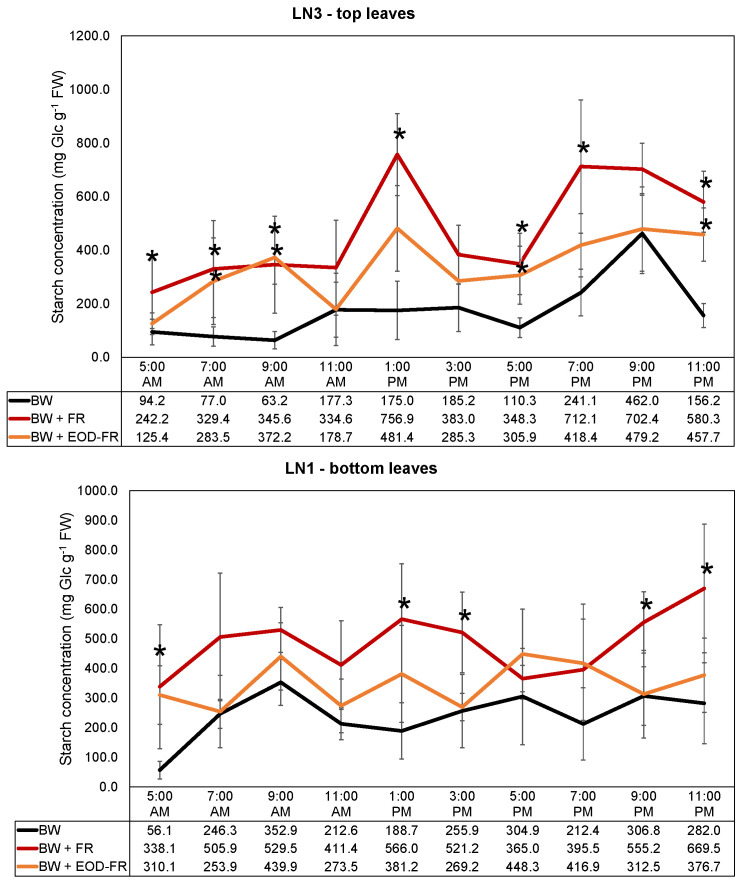
Daily evolution of the starch concentration (mg Glc g^−1^ FW) ± SD in top and bottom leaves of sweet basil plants grown under different light treatments. Asterisks (*) denote statistically significant effects of FR (BW + FR, in red) and EOD-FR (BW + EOD-FR, in orange) treatments compared to the control (BW, in black), as tested with a Tukey HSD test (*n* = 5, *p* < 0.05).

In Figure 4 and Table 2, the results for the starch measurements between 5:00 a.m. and 11:00 p.m. are displayed. The starch concentration in the top and bottom leaves was significantly higher for the treatment with additional FR light. Considering the relative increase (%) in the starch content, the addition of FR (BW + FR) increased the content significantly in the top leaves with 52% to 447%, and in the bottom leaves with 20% to 503% compared to BW treatment (Table 2), with averages of 220 ± 125% and 138 ± 137% for top and bottom leaves, respectively. Similar but smaller effects of the addition of FR were observed for the BW + EOD-FR treatment. However, the increases in the starch content compared to the BW treatment were still between 1 and 489% (top leaves) and 2 and 453% (bottom leaves), with average increments of 147 ± 151% and 80 ± 136% for top and bottom leaves, respectively. Strikingly, the percentual increases compared to BW were the highest at the beginning of the light period (Table 2). Considering absolute starch contents, the average starch content in the top leaves was significantly higher for the BW + FR (473.5 ± 192.9 mg Glc g^−1^ FW) and BW + EOD-FR (338.8 ± 124.6 mg Glc g^−1^ FW) treatments than for the BW treatment (174.1 ± 115.5 mg Glc g^−1^ FW). For the bottom leaves, the highest value was noted for BW + FR (485.7 ± 104.7 mg Glc g^−1^ FW), being significantly higher than the one for BW + EOD-FR (348.2 ± 73.4 mg Glc g^−1^ FW), which is itself significantly higher than the value for BW (241.9 ± 82.6 mg Glc g^−1^ FW). The starch concentration in the top leaves gradually increased for the BW + FR and BW + EOD-FR treatments, with a peak around 1:00 p.m., while for the BW treatment, it stayed fairly constant until 5:00 p.m., when it increased until the end of the light period (9:00 p.m.) (Figure 4). 

## 3. Discussion

### 3.1. Addition of FR Radiation Increases Biomass Production and Resource-Use Efficiency

Sweet basil plants show shade avoidance responses induced by additional FR in the light spectrum. This growth response is proven to be driven by changing the ratio of active to inactive phytochromes [37,38]. One of the most striking adaptations observed in dicotyledonous plants subjected to a low R:FR ratio is stem elongation [39,40]. In this study, plant height (Table 1) increased significantly under additional FR, with increases of 68% and 49% compared to BW. The stem-to-leaf ratio significantly increased when FR light was added to the spectrum (both BW + FR and BW + EOD-FR) as a consequence of higher stem biomass production. When FR light was supplied during the total photoperiod, this increased stem biomass production was not at the expense of leaf biomass formation, which was the case for BW + EOD-FR (Table 1).

Emerson et al. (1957) stated that the photosynthetic rate under simultaneous illumination with long (λ > 680 nm) and short wavelengths was greater than the sum of the photosynthetic rates obtained when illuminating plants with two light spectra separately [41]. They hypothesized that this synergistic effect on photosynthesis was the result of the enhancement of the quantum yield of longer wavelengths by shorter wavelengths, balancing the excitation of photosystems I and II, decreasing non-photochemical quenching, while increasing the net photosynthetic rate (Emerson Enhancement Effect). The other way around, Zhen and van Iersel (2017) added long wavelengths (735 nm) to shorter wavelengths, similarly resulting in positive effects on net photosynthesis, also potentially associated with the increased sink strength of tomato fruits [13,31]. Modeling approaches led to good predictive outcomes on the effects of R:FR ratios on the photosynthetic efficiency and potential plant biomass production [42]. Accordingly, by adding FR light during the total photoperiod (BW + FR), the fresh and dry weight of the leaves increased by 47% and 46%, respectively, while the fresh and dry weight of the stems increased even more spectacularly by 130% and 173%, respectively, in comparison with the control treatment (Table 1). An intermediate reaction was noted when adding FR at the end (last 2 h) of the photoperiod (BW + EOD-FR).

In addition to the effects on plant morphology, adding FR radiation to the light spectrum positively influenced the resource-use efficiency, more specifically, WUE and EUE (Figure 1). This combination of a high biomass yield under additional FR with an increased resource-use efficiency is very desirable for growers. The increased energy cost of including FR wavelengths [43] is more than compensated by a higher biomass production and WUE. This effect is especially significant for including FR during the total photoperiod (BW + FR), but it is also beneficial when using an end-of-day FR treatment (BW + EOD-FR).

The use of FR (701–750 nm) light in plant cultivation received considerable attention [35,44,45,46,47]. Indoor cultivation has gained popularity in recent years as environmental variables can be adjusted dynamically during the cultivation period on a crop-specific level [48,49]. However, as FR wavelengths are not included in the PAR definition, they are often forgotten in the manufacturing of LED lamps for crop production [46]. Recently, several researchers have recommended including FR photons (701–750 nm) in the definition of PAR, expanding it to ePAR (400–750 nm), as this definition better predicts photosynthesis [46,50]. Our results are in agreement with this proposal to extend the PAR definition as including FR light in the illumination spectrum provided substantial benefits in terms of biomass production and resource-use efficiency, both for BW + FR and BW + EOD-FR. Although both light spectra had similar PPFD values, the relative impacts of the FR photons on plant morphology, photosynthesis, and resource-use efficiency reflect their distinct mechanism for enhancing photosynthesis by improving the photochemical efficiency [50].

### 3.2. Additional FR Radiation Increased Carbohydrate Levels in Both Top and Bottom Leaves of Sweet Basil

Sugar metabolism and signaling are central in plant source–sink relationships and carbohydrate partitioning. As starch is osmotically inert, the plant perceives soluble sugars as signals, for instance, Glc, Suc, and trehalose-6-phosphate [51]. Based on the soluble sugars, determining carbon abundance or scarcity in that plant’s organ, a decision is made within the plant [6]. The partitioning of starch and Suc, the major storage carbohydrates, operates both on a short-term and long-term level. On a short-term level, it depends on the photosynthetic rate, while on a long-term level, the plant needs to store enough starch reserves to use during the coming night [6,8].

In this study, the soluble sugars Glc, Fru, Suc, Raf, Sta, and 1-K were measured in the top and bottom leaves of sweet basil. Glc was the most abundant in both top and bottom leaves, followed by Fru and Suc (Figure 2). Maltose was also found in some samples at very low concentrations between 0 and 0.20 mM (data not shown). Consistent with the FR effects on plant morphology, additional FR greatly influenced sugar concentrations in top and bottom leaves. The greatest effects of the addition of FR on sugar concentrations were observed in the BW + FR treatment. Due to the Emerson enhancement effect, the combination of FR with shorter wavelengths (BW + FR) results in a higher photosynthesis rate compared to the BW background alone [35,41]. Our results show increased biomass formation (Table 1), sugars (Figure 2 and Figure 3), and starch (Figure 4) accumulation under the addition of FR light, both in the top and bottom leaves, which supports the hypothesis of photosynthesis enhancement when FR light is supplied to the plants together with BW light. The distribution of sugars over the different plant organs (roots, flowers, leaves) is an important factor for plant survival and productivity. The overall absolute sugar concentration was higher in the top leaves compared to the bottom leaves for all three treatments (Figure 2). This supports the hypothesis that the top leaves are highly photosynthetically active, providing sugars for transport and growth (sugar sources). The higher sugar concentration in younger leaves may also be the consequence of a larger leaf surface area directly exposed to light, while older leaves are shaded by younger leaves.

Starch, the main storage carbohydrate in sweet basil leaves, increased significantly (up to 500%) in the top and bottom leaves under FR light addition for both BW + FR and BW + EOD-FR (Figure 4 and Table 2). This relative increase caused by additional FR was the highest in the morning (Table 2). Plants need to maintain carbon availability to maximize their growth. During the night, when photosynthesis is not possible, carbon should be supplied for metabolism and growth from stored carbon [52]. Therefore, the growth rate at night is highly dependent on the starch concentration. If the stored starch is depleted, the nightly growth rate will drop. Therefore, it is essential to build up a sufficiently large starch pool during the day to avoid the depletion of starch pools during the night, resulting in sugar starvation at the end of the dark period. The addition of FR to the light spectrum significantly increased the amount of starch stored at the beginning of the dark period (Figure 4). This increase was noted for both top and bottom leaves, providing opportunities for increased Sta export from the leaves during the night. This increase in the starch concentration in the top leaves of FR-treated plants was accompanied by an increase in the chlorophyll level of these top leaves (LN3) (Table 1), with increments of 23% (BW + FR) and 22% (BW + EOD-FR). These results show the increased starch-source strength and sugar-source of the top leaves, supplying the top leaves with large amounts of carbon for growth by being highly photosynthetically active.

Our results are consistent with Kasperbauer et al. (1970) [53], who investigated the effects of end-of-day FR radiation on the free sugars of tobacco leaves (*Nicotiana tabacum* L.). They found that EOD-FR radiation caused an increase in the concentrations of free sugars (Glc and Fru) and organic acids (malic, succinic, and fumaric acid) compared to EOD-R [53]. Lercari (1982) [54] found that reducing sugars (presumably Glc and Fru) accumulated in onion leaves (*Allium cepa* L. cv. Dorata di Parma) when the offered light was supplemented with FR, by mixing fluorescent lamps with incandescent lamps [54]. Kalaitzoglou et al. (2019) investigated whether there were differences in the responses of tomato fruit (*Solanum lycopersicum* ‘Komeett’) to additional FR light or EOD-FR light in the presence of broadband background radiation [35]. They found that the addition of FR light increased the tomato fruit yield, which correlated well with the overall increase in leaf source strength under FR.

It has been hypothesized that the carbon accumulation, both sugars and starch, in the leaves shows a phytochrome-mediated response [54,55]. Phytochromes can either be in an inactive (P_r_, absorption maximum at 660 nm) or active (P_fr_, absorption maximum at 730 nm) form. The ratio between the two forms dynamically changes with the composition of the light spectrum, strongly correlated with R:FR proportions in the light spectrum [56,57]. The addition of FR to the BW light spectrum decreased the R:FR ratio from 41.4 (BW) to 2.0 (BW + FR). We hypothesize that the observed increases in the concentration of the soluble sugars and starch in the top and bottom leaves of sweet basil are the result of both elevated photosynthesis, by the Emerson Enhancement effect and possibly also by stimulating phloem unloading in sink tissues, resulting in a more effective long-distance translocation of Sta to sinks, such as the stems [58]. The fact that additional FR, resulting in a lower R:FR ratio and thus more P_r_, stimulates unloading and long-distance transport has been reported only once in corn leaf strips (*Zea mays* L. cv. Golden Bantam) [58]. Moreover, the results were obtained for a monocot Suc translocator, which is physiologically different from a dicot Sta translocator. It is important to notice that we did not find more recent publications regarding the effects of FR light on the stimulation of phloem unloading. To formulate a more precise hypothesis regarding this effect, more research is needed to uncover the specific processes behind the effect of FR light both on phloem loading and unloading processes.

### 3.3. Suc Homeostasis and Sugar Transport

Sugar homeostasis is a dynamic process. In this process, starch and Suc undergo interconversion dependent on the cellular requirement [59]. A high starch concentration is an advantage to plants, which are sessile and hence unable to forage for food. For example, when the assimilation of carbohydrates becomes compromised, starch metabolism can buffer against carbon depletion [9]. Under additional FR light, the starch concentration significantly increased in the top and bottom leaves compared to the control spectrum (BW). Here, top and bottom leaves are considered source leaves, as they are fully expanded leaves capable of performing photosynthesis. The stem internodes and roots are considered carbohydrate sinks.

Sweet basil is an annual labiate, using RFOs, particularly Sta, as transport carbohydrates using the polymer trapping model, with Suc as the main precursor for RFO biosynthesis [28]. In the polymer trapping model, Suc diffuses from the mesophyll (bundle sheet cell) to the intermediary cell (IC) through highly branched plasmodesmata [60]. In these ICs, which are specialized companion cells of the phloem of minor veins, Raf and Sta are synthesized [26,61]. These RFOs are larger than Suc, hence unable to diffuse back to the mesophyll, generating high osmolarities for long-distance transport to sinks. In this study, it was found that Raf concentrations decreased during the morning and increased in the afternoon, with a minimum at 1:00 p.m. This pattern was not found under additional FR light, neither for Sta, nor for 1-K. This indicates that the bottleneck for Sta synthesis and subsequently phloem loading is situated at the level of RafS activity. FR enrichment of the light spectrum helped to overcome this early afternoon bottleneck.

**Figure 5:**
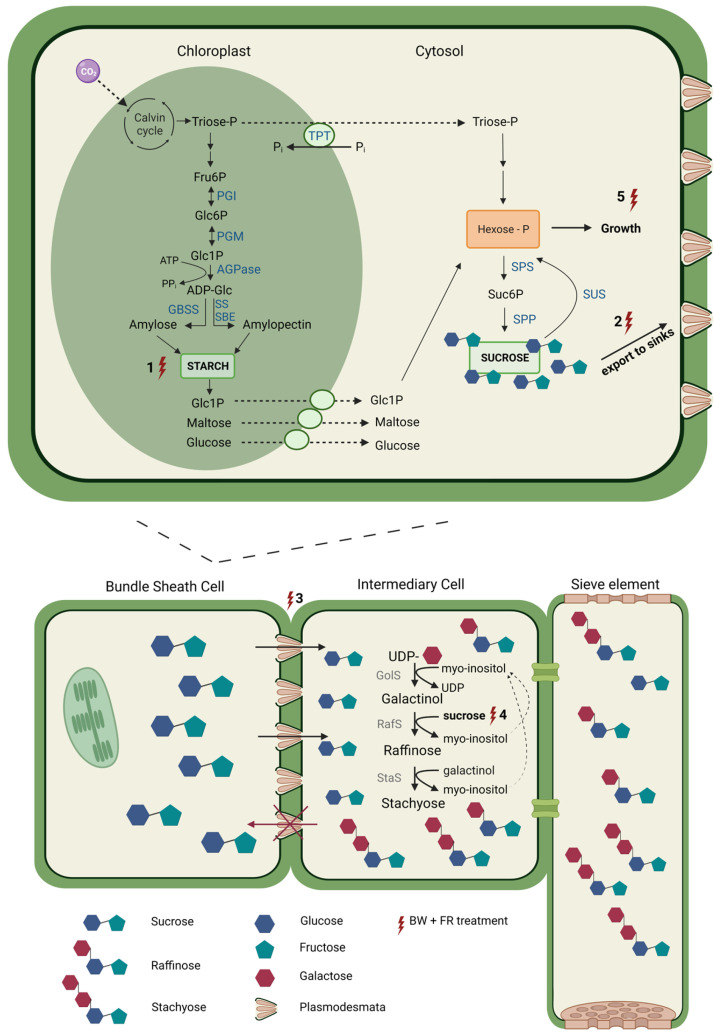
Schematic illustration of the increase in starch and small sugars pools in sweet basil source leaves under FR light treatment, potentially associated with more efficient stachyose production and phloem loading because of increased sucrose homeostasis in intermediary cells. Lightning bolts depict the different hypothetical influences of the addition of FR to the BW light treatment. TPT: triose phosphate/phosphate translocator; PGI: phosphoglucose isomerase; PGM: phosphoglucomutase; AGPase: ADP-glucose pyrophosphorylase; GBSS: granule-bound starch synthase; SBE: starch branching enzyme; SPS: sucrose phosphate synthase; SPP: sucrose phosphate phosphatase; SUS: sucrose synthase; GolS: galactinol synthase; RafS: raffinose synthase; StaS: stachyose synthase (Created with BioRender.com).

To ensure an increased yield, strong sugar homeostasis is required, and the presence of different versatile sugar pools is a plus. We hypothesize that adding FR light to the light spectrum significantly influences sugar homeostasis at many levels. Figure 5 displays an overview of where we hypothesize that additional FR light may influence this balance, depicted by the numbered lightning bolts. First, in the bundle sheet cells (the mesophyll), photosynthesis converts light energy into chemical energy in the form of Suc. Excess energy is stored in the chloroplasts as starch. The partitioning of photoassimilates between Suc and starch is highly regulated, with Triose-P (more specifically, 3-phosphoglyceric acid) being the key regulatory checkpoint [9]. Under carbon sufficiency, Triose-P is used to synthesize Suc for export to sinks and to stimulate starch synthesis. Suc export slows down, in case of reduced sink activity or reduced photosynthesis, and P_i_ increases in the chloroplast, leading to the inhibition of starch biosynthesis for storage. In the case of Suc depletion, starch is then broken down to release the stored carbon [9]. As seen in our data, adding FR light increases the starch concentration up to 500% in both leaf levels (both considered source leaves) compared to BW (Figure 5, number (1)). We hypothesize that in addition to the Emerson Enhancement Effect, hence increasing the photosynthetic rate, starch pools in sweet basil leaves are designed to contribute to Suc homeostasis in a highly regulated manner under additional FR light. In normal circumstances, the rate of starch degradation at night is approximately linear in such a way that 95% of the starch is used at the start of the next light period, hence 5% will be left [52]. When calculating this for treatment BW, 20% (LN3, top leaves) and 18% (LN1, bottom leaves) of the starch was left at the start of the light period (comparison between 9:00 p.m. and 5:00 a.m.) (Figure 4). However, for BW + FR, 34% (LN3, top leaves) and 61% (LN1, bottom leaves) of the starch were left at the start of the light period/end of the dark period. We hypothesize that starch pools under additional FR have a broader function than solely providing carbon during the night. They may also contribute to a strong sugar homeostasis in the source leaves able to provide a steady input of carbon for growth and export during the whole diurnal cycle and even under temporal stress situations (Figure 5, numbers (5) and (2)). Therefore, it can be hypothesized that plants greatly benefit from the increment of starch pools in source leaves under additional FR light.

Additionally, increasing the available carbohydrate pools offers opportunities to increase sugar export to sinks such as stems, roots, and developing sink leaves (Figure 5, numbers (2) and (3)). Once Suc is diffused to the ICs, it serves as the main precursor (together with Gol) for the synthesis of Raf and Sta, the latter being the main transport sugar in sweet basil [28] (Figure 5, number (4)). The interplay of the three enzymes (GolS, RafS, and StaS) only works perfectly when a constant supply of Suc can be provided to RafS. If not, RafS will hydrolyze Gol [29]. Plants cultivated under BW + FR not only show a significant increase in Suc in top and bottom leaves, but the Suc concentration also remained more constant during the day (Figure 2). Unlike the plants of BW, showing a decrease in the Suc concentration around 1:00 p.m., BW + FR-treated plants show a constant Suc concentration. Similarly, the Raf concentration also increased under BW + FR in the early afternoon (Figure 3), mainly for the bottom leaves, avoiding Raf depletion and the production of Sta for continued phloem loading. Moreover, both BW + FR and BW + EOD FR were able to counteract the decrease in Sta at the beginning of the dark periods, suggesting that these plants may be superior in sustaining continued nocturnal Sta export from the leaves. However, this requires further verification.

Additionally, BW + FR plants did not show a strong morning Fru peak (Figure 2, LN3) compared to BW plants, while the BW + EOD-FR treatment partially reduced the Fru peak. Although the origin of this peak is unclear, different hypotheses can be raised. In research on *Arabidopsis*, increased SnRK1 activity has been observed during the night while starch decreased [51]. Right before dawn, this may cause a sugar starvation response. Interactome studies have suggested that the cytosolic invertase CINV1 is an interaction partner from SnRK1 [62], potentially phosphorylating CINV1. The transition of dark to light stimulates the binding of 14-3-3 to the phosphorylated CINV, leading to its activation [63] and degradation of cytosolic Suc to Glc and Fru. Typically, Glc is metabolized more quickly, while Fru may persist for a while. It can be hypothesized that FR light prevents SnRK1 activation and CINV phosphorylation due to higher residual sugar and starch levels right before dawn. Alternatively, or additionally, FR light may contribute to the phosphorylation of Fru to Fru 2,6-P_2_ (fructose 2,6 biphosphate), a central regulator in the partitioning of photosynthate between Suc and starch [10]. Fru 2,6-P_2_ contributes to the control of photosynthetic Suc feed-forward. Feed-forward control comprises the coordination of the Suc synthesis rate with the ability to supply triose-P during photosynthesis by the chloroplast. The feedback control comprises the conversion from Suc to starch when the Suc synthesis rate exceeds the ability of the leaf to export it or store it in the cytosol [10]. Hence, from the observation that the BW + FR-treated plants do not exhibit this Fru peak, it is hypothesized that FR light influences the Fru phosphorylation process, potentially leading to a more efficient cellular Fru homeostasis and/or more efficient crosstalk between the different carbon pools. These ideas offer great avenues for future work.

### 3.4. EOD-FR Causes an Intermediate Effect Compared to Constant FR Addition

Far-red supplementation can be applied near the end-of-day to mimic the increase in FR in the light spectrum under natural light conditions (BW + EOD-FR). These EOD-FR treatments can be used as an energy-saving strategy to influence plant development, either in greenhouses or indoor cultivation [64]. However, the EOD-FR treatments were found to be less effective than BW + FR in studies on tomato [35] and *Chenopodium album* L. [65]. Our results are in line with these reports, both on a plant morphological level and at the level of the starch concentration and amount of soluble sugars. The morphological differences induced by the addition of FR were more pronounced when the treatment was administered during the 16 h photoperiod rather than in the last 2 h of the photoperiod.

While EOD-FR has been shown to increase the light energy use efficiency, it did not have the same impact on EUE as BW + FR: EUE increased by 40% (BW + FR) on a fresh weight basis, while BW + EOD-FR showed an increase of 28%. EOD-FR has been proposed as an alternative use of supplemental FR, hypothesizing that it is more energy efficient while still evoking the shade avoidance response [34,35,36]. In our study, it was indeed more energy efficient than the control treatment, but the provided additional FR during the total photoperiod (BW + FR) was found to increase the EUE even further. WUE was increased similarly for both treatments involving FR light, with increases of 30% and 34% on a fresh weight basis compared to the control treatment for the BW + FR and BW + EOD-FR treatments, respectively.

Differences in the sugar and starch content compared to the control treatment, associated with additional FR, were more pronounced when the treatment was administered during the 16 h photoperiod (BW + FR) rather than during the last 2 h of the photoperiod (BW + EOD-FR). Our results are consistent with Ranwala et al. (2002), who investigated changes in the soluble carbohydrate concentration in leaves of watermelon (*Citrullus lanatus* Thunb. Matsum and Nakai cv. Sugar Baby) seedlings. They found that 15 min of 68 µmol m^−2^ s^−1^ of FR light at the end of the light period increased the total soluble carbohydrate concentration in leaves, with significant effects on the Glc and Fru concentrations [66]. In our results, significant increases were found at 7:00 a.m. and 9:00 a.m. (Glc, top leaves), 7:00 a.m. (Suc, top leaves), 5:00 p.m. (Glc, bottom leaves), and 11:00 a.m. (Suc, bottom leaves), and at 7:00 a.m., 9:00 a.m., 5:00 p.m., and 11:00 p.m. (starch, top leaves) (Figure 2 and Figure 3).

## 4. Materials and Methods

### 4.1. Growth Conditions and Light Treatments

The experiment was conducted in 3 growth chambers, with sweet basil plants (*Ocimum basilicum* L. cv. Gustosa) grown in plant containers filled with a commercial soil substrate. Plants were cultivated similarly to Schenkels et al. (2020), with 35 plant containers per growth chamber, with 19 ± 4 plants per container. The climate in the growth chambers was controlled to obtain a 16 h photoperiod/8 h dark period, 24 °C day temperature, 18 °C night temperature, and 70% relative humidity [67]. The light treatments were initiated immediately after placing the plant containers in their respective growth chambers. The light intensity was kept at around 125 µmol m^−2^ s^−1^ at the plant level. Two multispectral LED lamps were placed 100 cm above plant level. Three different treatments were investigated: (1) broad white light spectrum (40–700 nm) during the 16 h photoperiod (=‘BW’), (2) BW with FR addition during the 16 h photoperiod (=‘BW + FR’), and (3) BW during the first 14 h of the photoperiod, followed by the BW + FR light spectrum in the last 2 h of the photoperiod (=‘BW + EOD-FR’) (EOD, end-of-day*,* referring to the end of the light period). The relative share of different wavebands (%) and the PPFD at the plant level (photosynthetic photon flux density, µmol m^−2^ s^−1^) are presented in Table 3. 

### 4.2. Biometric Measurements 

After 37 days of sowing, 10 plant containers per treatment were harvested. The chlorophyll level (SPAD value) (Soil Plant Analysis Development, MC-100, Apogee Instruments, Inc., Logan, UT, USA) was measured on an area between the central vein and the leaf edge, avoiding the major leaf vein, at LN1 and LN3 (BBCH scale 14, 4th true leaf unfolded). The average plant height (cm) was determined by measuring the 3 longest stems per plant container. The leaves and stems were weighed separately to determine the fresh weight (FW, in g) and were subsequently placed in a ventilated drying oven (ICP500, Memmert GmbH + Co. KB., Büchenbach, Germany) at 70 °C for 7 days to determine the dry weight (DW, in g). The amount of water transpired (mL) was calculated from the soil moisture sensor (Soil Pro Mini, Sigrow, Ede, the Netherlands) data, as described [68]. With these data, the water use efficiency (WUE, g FW/DW L^−1^) per plant container could be calculated as the ratio of the yield of leaves and stems (g, fresh or dry) over the amount of transpired water (L). The nutrient use efficiency (NUE, g FW/DW kg^−1^ nutrients) was calculated as the ratio of FW or DW of the leaves and stems over the amount of nutrients taken up per plant container. The amount of nutrients taken up from the soil by the plant was calculated using the soil moisture sensors, which also measured the soil electrical conductivity (mS cm^−1^). A linear relationship was found between the sensors’ measurement of electrical conductivity (mS cm^−1^) and the fertilizer concentration in the substrate (FC) (g L^−1^): EC (mS cm^−1^) = 0.1952 FC (g L^−1^) + 0.0087 (R^2^ = 0.99, *n* = 12). The cumulated nutrient uptake per plant container was calculated for the experimental period. The electrical energy use efficiency (EUE, in g FW/DW W^−1^ m^2^) was calculated as the ratio of the fresh/dry yield of leaves and stems (g) over the lamps’ cumulated electricity consumption (W m^−2^), calculated for each light spectrum separately. The average fresh and dry yield of leaves and stems per treatment was used for the calculation of EUE.

### 4.3. Sugar and Starch Measurements

At the end of the cultivation period (37 days after sowing), top (LN 3) and bottom (LN 1) leaves of a similar size were flash-frozen in liquid nitrogen. Leaf sampling was performed, starting at 5:00 a.m. (one hour before the lights were put on) until 11:00 p.m. (one hour after the lights were switched off) with two-hour intervals. Per sampling time, five samples of the top leaves and five samples of the bottom leaves were collected, containing four leaves per sample divided over three plant containers. At the end of the sampling period, a total of 300 samples were collected. These samples were ground with a mortar and pestle to a fine, homogenous powder for further sugar and starch extractions. Samples of different sampling times, treatments, and leaf levels were analyzed in a random order. The extraction of soluble sugars and starch was performed following the procedure outlined in [69]. Then 3 volumes of 400 µL of 80% EtOH-ddH_2_O solution (50 °C) were added to 100 mg of frozen plant material in 3 consecutive steps. After each step, the plant material was heated for 5 min at 82 °C and crushed with a plastic pestle before centrifugation at 14,000 rpm for 5 min. The supernatant was removed and collected after every step. The supernatant was used in the soluble sugar analysis, while the pellet was used for the starch extraction. The supernatant was vacuum-centrifuged to allow EtOH to evaporate and was afterward redissolved in 10 volumes of ddH_2_O. After vortex and centrifugation at 14,000 rpm for 5 min, 200 µL of supernatant was transferred to a Dowex^®^ column containing H^+^ and Ac^−^ resins (Sigma-Aldrich, Burlington, MI, USA). Next, the column was washed 6 times with 200 µL of ddH_2_O. The total amount of 1200 µL flowthrough was collected and centrifuged at 14,000 rpm for 5 min. The samples were then analyzed using high-performance anion-exchange chromatography with integrated pulsed amperometric detection (HPAEC-IPAD; ICS-3000, Dionex, ThermoFisher Scientific, Waltham, MA, USA) to determine the contents of the soluble sugars Glc, Fru, Suc, Raf, Sta, and 1-K. For the starch extraction, the pellet was boiled at 100 °C for 10 min in 500 µL of 0.02% NaN_3_, after which the samples were centrifuged for 5 min at 14,000 rpm. The supernatant (100 µL) was used to set up the reaction for starch degradation in 1200 µL of NaOAc buffer (50 mM, pH 5.0). To degrade the starch, 50 µL of α-amylase and 2.5 mg of amyloglucosidase in 250 µL of ddH_2_O were added to this buffer. The reactions were set up overnight at 30 °C in 30 µL of NaAc (1 M, pH 5.0) and 20 µL of the NaOAc buffer, and afterward they were halted by boiling the samples at 100 °C for 5 min. The enzymes were deactivated by boiling the samples (10 µL) for 5 min at 100 °C before being added to the samples to form the blank controls. Blank controls and overnight samples were analyzed using HPAEC-IPAD. The starch content is expressed in mg Glc g^−1^ FW.

### 4.4. Statistical Analysis

The Shapiro–Wilk test was used to test whether the measured variables were normally distributed. If the assumption of normality was met, a Tukey HSD test was used to compare the means of the different treatments (*p* < 0.05). The non-parametric Wilcoxon rank sum test was used if the normality assumption was not fulfilled. Statistical analyses were performed using JMP Pro 16 (SAS Institute Inc., Cary, NC, USA). Graphs were generated using Prism (GraphPad Software, San Diego, California, USA) and Microsoft Excel.

## 5. Conclusions and Perspectives

The inclusion of FR light in the light spectrum produced sweet basil plants with an increased biomass yield, stem elongation, resource-use efficiency, and strongly increased carbohydrate concentrations in the leaves. Although these observations were less prominent when FR was only added during the last 2 h before the dark period, this treatment is very cost-effective, providing additional opportunities for the more efficient commercial indoor production of sweet basil [32]. However, it should be noted that extra illumination may induce discrete suites of volatile classes that affect sensory quality in commercial herbs, which requires a careful evaluation before commercial exploitation [70].

Through extended sugar analyses, with a focus on the starch and RFO pools, we obtained insights that will boost further studies optimizing FR light treatments in sweet basil and other plant species. In particular, we found that the addition of FR light to a BW spectrum increased the sugar and starch concentrations in the top and bottom leaves of young sweet basil plants. Adding FR during the total photoperiod (BW + FR) had a larger effect on the leaf sugar and starch concentration than the addition of FR at the end of the day (BW + EOD-FR). We hypothesized that the increase in sugar and starch concentrations in the top and bottom leaves of sweet basil under the addition of FR light may be the result of both elevated photosynthesis through the Emerson effect in source leaves and increased and/or more stable Sta export with the help of more extended and versatile starch pools.

In terms of further increasing our mechanistic understanding of the physiological processes that occur under FR light addition in sweet basil, we suggest two main research directions: (i) further research should be conducted into phloem unloading in stem internodes provided with sugars by adjacent source leaves, and (ii) further studies on the export of Sta and various plant hormones during the dark period as well as during the light period. Referring to the hormonal context, a fast and efficient non-polar transport of auxin in the phloem of *Coleus blumei* was reported [71]*,* a close relative to sweet basil, occurring at a speed that is at least one order of magnitude faster than the polar auxin transport in *Arabidopsis* [17,72]. Therefore, it can be hypothesized that the rapid phloem-mediated co-transport of auxin and Sta could be possible in sweet basil, contrary to *Arabidopsis*, which is an apoplastic loader that does not make use of RFOs as transport sugars by polymer trapping. As such, the import of leaf-born auxin into the stems may rapidly stimulate stem elongation, diminishing the requirement for local auxin synthesis. The differentiation of phloem in stems highly depends on auxin, although the auxin/gibberellin ratio is crucial [73]. Upon phloem unloading, Sta needs to be degraded to hexoses by a combination of α-galactosidase [74] and invertase activities [16] contributing to sink strength. Accordingly, FR light was reported to increase the activity of plant invertases [75]. Moreover, recent discoveries highlighted intense crosstalk between phytochrome interacting factors (PIFs) and sugar metabolism and signaling in a circadian context [76,77,78], urging further research into PIFs in sweet basil in particular and in RFO-transporting plants in general.

## Figures and Tables

**Table 1:** Effect of additional far-red radiation during the total photoperiod (BW + FR) or in the last 2 h of the photoperiod (BW + EOD-FR) on plant height (cm), chlorophyll level (SPAD value) of LN1 (bottom leaves) and LN3 (top leaves), fresh weight of leaves and stems (g), dry weight of leaves and stems (g), and leaf-to-stem ratio ± SD. Letters show significant differences between the treatments, according to the Tukey HSD test (*p* < 0.05, *n* = 10).

Parameter	Units	BW		BW + FR		BW + EOD-FR	
Plant height	cm	7.8 ± 1.0	B	13.1 ± 2.1	A	11.6 ± 1.7	A
Chlorophyll level LN1	SPAD	29.9 ± 2.9	A	27.6 ± 4.2	A	31.1 ± 3.4	A
Chlorophyll level LN3	SPAD	23.1 ± 0.2	B	28.5 ± 3.6	A	28.1 ± 2.5	A
Fresh weight leaves	g	11.2 ± 2.2	B	16.5 ± 1.7	A	12.7 ± 4.1	B
Fresh weight stems	g	2.7 ± 0.6	C	6.1 ± 1.1	A	4.7 ± 1.0	B
Dry weight leaves	g	0.8 ± 0.2	B	1.2 ± 0.1	A	0.9 ± 0.3	B
Dry weight stems	g	0.2 ± 0.0	C	0.4 ± 0.1	A	0.3 ± 0.1	B
Leaf-to-stem ratio (fresh)	%	4.3 ± 0.8	A	2.8 ± 0.7	B	2.8 ± 0.9	B
Leaf-to-stem ratio (dry)	%	5.4 ± 1.3	A	3.0 ± 0.8	B	3.1 ± 1.1	B

**Table 2:** Relative increase (%) in starch content in the top and bottom leaves of sweet basil plants cultivated under additional FR (BW + FR and BW + EOD-FR) compared to a broad white light spectrum without FR light (BW). The percentual increments are color-coded depending on the order of magnitude.

Time	Relative Increase (%) in Starch Content Compared to BW				
	Bottom Leaves (LN1)			Top Leaves (LN3)	
	BW + FR	BW + EOD-FR		BW + FR	BW + EOD-FR
5:00 a.m.	503	453		157	33
7:00 a.m.	105	3		328	268
9:00 a.m.	50	25		447	489
11:00 a.m.	93	29		89	1
1:00 p.m.	200	102		333	175
3:00 p.m.	104	5		107	54
5:00 p.m.	20	47		216	177
7:00 p.m.	86	96		195	74
9:00 p.m.	81	2		52	4
11:00 p.m.	137	34		271	193
Color legend	0–100	101–200	201–300	301–400	>400

**Table 3:** Main features of the light spectra used in the experiment. Photosynthetic photon flux density (PPFD) and spectral properties are reported.

	PPFD (µmol m^−2^ s^−1^)	The Relative Share of Different Wavebands (%)			
		400–500 nm	501–600 nm	601–700 nm	701–800 nm
BW	125.4	29	39	31	1
BW + FR	126.0	25	33	28	14

## Data Availability

Data can be obtained from the corresponding author upon request.

## References

[1] Lee S., Umano K., Shibamoto T., Lee K. (2005). Identification of volatile components in basil (*Ocimum basilicum* L.) and thyme leaves (*Thymus vulgaris* L.) and their antioxidant properties. Food Chem..

[2] Aminian A.R., Mohebbati R., Boskabady M.H. (2022). The Effect of *Ocimum basilicum* L. and Its Main Ingredients on Respiratory Disorders: An Experimental, Preclinical, and Clinical Review. Front. Pharmacol..

[3] Couée I., Sulmon C., Gouesbet G., El Amrani A. (2006). Involvement of soluble sugars in reactive oxygen species balance and responses to oxidative stress in plants. J. Exp. Bot..

[4] Halford N.G., Curtis T.Y., Muttucumaru N., Postles J., Mottram D.S. (2011). Sugars in crop plants. Ann. Appl. Biol..

[5] Sheen J., Zhou L., Jang J. (1999). Sugars as signaling molecules. Curr. Opin. Plant Biol..

[6] MacNeill G.J., Mehrpouyan S., Minow M.A.A., Patterson J.A., Tetlow I.J., Emes M.J. (2017). Starch as a source, starch as a sink: The bifunctional role of starch in carbon allocation. J. Exp. Bot..

[7] Bihmidine S., Hunter C.T., Johns C.E., Koch K.E., Braun D.M. (2013). Regulation of assimilate import into sink organs: Update on molecular drivers of sink strength. Front. Plant Sci..

[8] Smith A.M., Stitt M. (2007). Coordination of carbon supply and plant growth. Plant Cell Environ..

[9] Dong S., Beckles D.M. (2019). Dynamic changes in the starch-sugar interconversion within plant source and sink tissues promote a better abiotic stress response. J. Plant Physiol..

[10] Stitt M., Kurzel B., Heldt H.W. (1984). Control of Photosynthetic Sucrose Synthesis by Fructose 2,6-Bisphosphate—II. Partitioning between Sucrose and Starch. Plant Physiol..

[11] Smith A.M., Zeeman S.C. (2020). Starch: A Flexible, Adaptable Carbon Store Coupled to Plant Growth. Annu. Rev. Plant Biol..

[12] Thalmann M., Santelia D. (2017). Starch as a determinant of plant fitness under abiotic stress. New Phytol..

[13] Ji Y., Nuñez Ocaña D., Choe D., Larsen D.H., Marcelis L.F.M., Heuvelink E. (2020). Far-red radiation stimulates dry mass partitioning to fruits by increasing fruit sink strength in tomato. New Phytol..

[14] Li Y., Xin G., Wei M., Shi Q., Yang F., Wang X. (2017). Carbohydrate accumulation and sucrose metabolism responses in tomato seedling leaves when subjected to different light qualities. Sci. Hortic..

[15] Deng K., Yu L., Zheng X., Zhang K., Wang W., Dong P., Zhang J., Ren M. (2016). Target of rapamycin is a key player for auxin signaling transduction in arabidopsis. Front. Plant Sci..

[16] Ruan Y.L. (2014). Sucrose metabolism: Gateway to diverse carbon use and sugar signaling. Annu. Rev. Plant Biol..

[17] Yuan X., Xu P., Yu Y., Xiong Y. (2020). Glucose-TOR signaling regulates PIN2 stability to orchestrate auxin gradient and cell expansion in Arabidopsis root. Proc. Natl. Acad. Sci. USA.

[18] Morkunas I., Borek S., Formela M., Ratajczak L., Chang C.-F. (2012). Plant Responses to Sugar Starvation. Carbohydrates—Comprehensive Studies on Glycobiology and Glycotechnology.

[19] Rolland F., Baena-Gonzalez E., Sheen J. (2006). Sugar sensing and signaling in plants: Conserved and novel mechanisms. Annu. Rev. Plant Biol..

[20] Vu D.P., Rodrigues C.M., Jung B., Meissner G., Klemens P.A.W., Holtgräwe D., Fürtauer L., Nägele T., Nieberl P., Pommerrenig B. (2020). Vacuolar sucrose homeostasis is critical for plant development, seed properties, and night-time survival in Arabidopsis. J. Exp. Bot..

[21] Margalha L., Confraria A., Baena-González E. (2019). SnRK1 and TOR: Modulating growth–defense trade-offs in plant stress responses. J. Exp. Bot..

[22] Nägele T., Henkel S., Hörmiller I., Sauter T., Sawodny O., Ederer M., Heyer A.G. (2010). Mathematical modeling of the central carbohydrate metabolism in arabidopsis reveals a substantial regulatory influence of vacuolar invertase on whole plant carbon metabolism. Plant Physiol..

[23] De Coninck B., Le Roy K., Francis I., Clerens S., Vergauwen R., Halliday A.M., Smith S.M., Van Laere A., Van den Ende W. (2005). Arabidopsis AtcwINV3 and 6 are not invertases but are fructan exohydrolases (FEHs) with different substrate specificities. Plant Cell Environ..

[24] Van den Ende W. (2013). Multifunctional fructans and raffinose family oligosaccharides. Front. Plant Sci..

[25] Sengupta S., Mukherjee S., Basak P., Majumder A.L. (2015). Significance of galactinol and raffinose family oligosaccharide synthesis in plants. Front. Plant Sci..

[26] Turgeon R., Gowan E. (1990). Phloem loading in *Coleus blumei* in the absence of carrier-mediated uptake of export sugar from the apoplast. Plant Physiol..

[27] Madore M.A. (1990). Carbohydrate metabolism in photosynthetic and nonphotosynthetic tissues of variegated leaves of *Coleus blumei* Benth. Plant Physiol..

[28] Buchi R., Bachmann M., Keller F. (1998). Carbohydrate metabolism in source leaves of sweet basil (*Ocimum basilicum* L.), a starch-storing and stachyose-translocating labiate. J. Plant Physiol..

[29] Sprenger N., Keller F. (2000). Allocation of raffinose family oligosaccharides to transport and storage pools in *Ajuga reptans*: The roles of two distinct galactinol synthases. Plant J..

[30] Li T., Zhang Y., Liu Y., Li X., Hao G., Han Q., Dirk L.M.A., Downie A.B., Ruan Y.L., Wang J. (2020). Raffinose synthase enhances drought tolerance through raffinose synthesis or galactinol hydrolysis in maize and Arabidopsis plants. J. Biol. Chem..

[31] Zhen S., van Iersel M.W. (2017). Far-red light is needed for efficient photochemistry and photosynthesis. J. Plant Physiol..

[32] Rahman M.M., Vasiliev M., Alameh K. (2021). LED illumination spectrum manipulation for increasing the yield of sweet basil (*Ocimum basilicum* L.). Plants.

[33] Hogewoning S.W., Wientjes E., Douwstra P., Trouwborst G., van Ieperen W., Croce R., Harbinson J. (2012). Photosynthetic quantum yield dynamics: From photosystems to leaves. Plant Cell.

[34] Lanoue J., Little C., Hao X. (2022). The Power of Far-Red Light at Night: Photomorphogenic, Physiological, and Yield Response in Pepper During Dynamic 24 Hour Lighting. Front. Plant Sci..

[35] Kalaitzoglou P., van Ieperen W., Harbinson J., van der Meer M., Martinakos S., Weerheim K., Nicole C.C.S., Marcelis L.F.M. (2019). Effects of continuous or end-of-day far-red light on tomato plant growth, morphology, light absorption, and fruit production. Front. Plant Sci..

[36] Islam M.A., Liu J., Blystad D., Gislerød H.R., Torre S., Olsen J.E. (2014). Impact of end-of-day red and far-red light on plant morphology and hormone physiology of poinsettia. Sci. Hortic..

[37] Smith H., Whitelam G.C. (1997). The shade avoidance syndrome: Multiple responses mediated by multiple phytochromes. Plant Cell Environ..

[38] Franklin K.A. (2008). Shade avoidance. New Phytol..

[39] Ruberti I., Sessa G., Ciolfi A., Possenti M., Carabelli M., Morelli G. (2012). Plant adaptation to dynamically changing environment: The shade avoidance response. Biotechnol. Adv..

[40] Morgan D.C., Smith H. (1981). Control of Development in Chenopodium Album L. By Shadelight: The Effect of Light Quality (Red:Far-Red Ratio) on Morphogenesis. New Phytol..

[41] Emerson R., Chalmers R., Cederstrand C. (1957). Some Factors Influencing the Long-Wave Limit of Photosynthesis. Proc. Natl. Acad. Sci. USA.

[42] Murakami K., Matsuda R., Fujiwara K. (2018). A mathematical model of photosynthetic electron transport in response to the light spectrum based on excitation energy distributed to photosystems. Plant Cell Physiol..

[43] Kozai T. (2013). Resource use efficiency of closed plant production system with artificial light: Concept, estimation and application to plant factory. Proc. Jpn. Acad. Ser. B Phys. Biol. Sci..

[44] Kono M., Kawaguchi H., Mizusawa N., Yamori W., Suzuki Y., Terashima I. (2020). Far-red light accelerates photosynthesis in the low-light phases of fluctuating light. Plant Cell Physiol..

[45] Park Y., Runkle E.S. (2017). Far-red radiation promotes growth of seedlings by increasing leaf expansion and whole-plant net assimilation. Environ. Exp. Bot..

[46] Zhen S., van Iersel M., Bugbee B. (2021). Why Far-Red Photons Should Be Included in the Definition of Photosynthetic Photons and the Measurement of Horticultural Fixture Efficacy. Front. Plant Sci..

[47] Zhen S., Bugbee B. (2020). Substituting Far-Red for Traditionally Defined Photosynthetic Photons Results in Equal Canopy Quantum Yield for CO_2_ Fixation and Increased Photon Capture during Long-Term Studies: Implications for Re-Defining PAR. Front. Plant Sci..

[48] SharathKumar M., Heuvelink E., Marcelis L.F.M. (2020). Vertical Farming: Moving from Genetic to Environmental Modification. Trends Plant Sci..

[49] Van Delden S.H., SharathKumar M., Butturini M., Graamans L.J.A., Heuvelink E., Kacira M., Kaiser E., Klamer R.S., Klerkx L., Kootstra G. (2021). Current status and future challenges in implementing and upscaling vertical farming systems. Nat. Food.

[50] Zhen S., Bugbee B. (2020). Far-red photons have equivalent efficiency to traditional photosynthetic photons: Implications for redefining photosynthetically active radiation. Plant Cell Environ..

[51] Avidan O., Moraes T.A., Mengin V., Feil R., Rolland F., Stitt M., Lunn J.E. (2022). Diel fluctuations in *in-vivo* SnRK1 activity in Arabidopsis rosettes during light-dark cycles. bioRxiv.

[52] Graf A., Smith A.M. (2011). Starch and the clock: The dark side of plant productivity. Trends Plant Sci..

[53] Kasperbauer M.J., Tso T.C., Sorokin T.P. (1970). Effects of end-of-day red and far-red radiation on free sugars, organic acids and amino acids of tobacco. Phytochemistry.

[54] Lercari B. (1982). The effect of far-red light on the photoperiodic regulation of carbohydrate accumulation in *Allium cepa* L. Physiol. Plant..

[55] Kasperbauer M.J., Hunt P.G., Sojka R.E. (1984). Photosynthate partitioning and nodule formation in soybean plants that received red or far-red light at the end of the photosynthetic period. Plant Physiol..

[56] Demotes-Mainard S., Péron T., Corot A., Bertheloot J., Le Gourrierec J., Pelleschi-Travier S., Crespel L., Morel P., Huché-Thélier L., Boumaza R. (2016). Plant responses to red and far-red lights, applications in horticulture. Environ. Exp. Bot..

[57] Franklin K.A., Whitelam G.C. (2005). Phytochromes and shade-avoidance responses in plants. Ann. Bot..

[58] Hoddinott J. (1983). the Influence of Light Quality on Carbohydrate Translocation within Corn Leaf Strips. New Phytol..

[59] Saddhe A.A., Manuka R., Penna S. (2021). Plant sugars: Homeostasis and transport under abiotic stress in plants. Physiol. Plant..

[60] Yadav U.P., Ayre B.G., Bush D.R. (2015). Transgenic approaches to altering carbon and nitrogen partitioning in whole plants: Assessing the potential to improve crop yields and nutritional quality. Front. Plant Sci..

[61] Rennie E.A., Turgeon R. (2009). A comprehensive picture of phloem loading strategies. Proc. Natl. Acad. Sci. USA.

[62] Van Leene J., Eeckhout D., Gadeyne A., Matthijs C., Han C., De Winne N., Persiau G., Van De Slijke E., Persyn F., Mertens T. (2022). Mapping of the plant SnRK1 kinase signalling network reveals a key regulatory role for the class II T6P synthase-like proteins. Nat. Plants.

[63] Gao J., Van Kleeff P.J.M., Oecking C., Li K.W., Erban A., Kopka J., Hincha D.K., De Boer A.H. (2014). Light modulated activity of root alkaline/neutral invertase involves the interaction with 14-3-3 proteins. Plant J..

[64] Zhen S., Kusuma P., Bugbee B. (2022). Toward an Optimal Spectrum for Photosynthesis and Plant Morphology in LED-Based Crop Cultivation.

[65] Morgan D.C., Smith H. (1978). The relationship between phytochrome-photoequilibrium and Development in light grown *Chenopodium album* L. Planta.

[66] Ranwala N.K.D., Decoteau D.R., Ranwala A.P., Miller W.B. (2002). Changes in soluble carbohydrates during phytochrome-regulated petiole elongation in watermelon seedlings. Plant Growth Regul..

[67] Schenkels L., Saeys W., Lauwers A., De Proft M.P. (2020). Green light induces shade avoidance to alter plant morphology and increases biomass production in *Ocimum basilicum* L. Sci. Hortic..

[68] Driesen E., De Proft M., Saeys W. (2021). Soil moisture levels affect the anatomy and mechanical properties of basil stems (*Ocimum basilicum* L.). Plants.

[69] Versluys M., Toksoy Öner E., Van den Ende W. (2022). Fructan oligosaccharide priming alters apoplastic sugar dynamics and improves resistance against *Botrytis cinerea* in chicory. J. Exp. Bot..

[70] Carvalho S.D., Schwieterman M.L., Abrahan C.E., Colquhoun T.A., Folta K.M. (2016). Light quality dependent changes in morphology, antioxidant capacity, and volatile production in sweet basil (*Ocimum basilicum*). Front. Plant Sci..

[71] Goldsmith M.H.M., Cataldo D.A., Karn J., Brenneman T., Trip P. (1974). The rapid non-polar transport of auxin in the phloem of intact Coleus plants. Planta.

[72] Ma L., Li G. (2019). Auxin-dependent cell elongation during the shade avoidance response. Front. Plant Sci..

[73] Aloni R. (1979). Role of Auxin and Gibberellin in Differentiation of Primary Phloem Fibers. Plant Physiol..

[74] Liu H., Liu X., Zhao Y., Nie J., Yao X., Lv L., Yang J., Ma N., Guo Y., Li Y. (2022). Alkaline α-galactosidase 2 (CsAGA2) plays a pivotal role in mediating source–sink communication in cucumber. Plant Physiol..

[75] Coffman C.M. (2016). Effect of Far-Red Induced Shade-Avoidance Responses on Carbon Allocation in *Arabidopsis Thaliana*. Ph.D. Thesis.

[76] Bolouri Moghaddam M.R., Van den Ende W. (2013). Sweet immunity in the plant circadian regulatory network. J. Exp. Bot..

[77] Bolouri Moghaddam M.R., Van den Ende W. (2013). Sugars, the clock and transition to flowering. Front. Plant Sci..

[78] Yang D., Liu Y., Ali M., Ye L., Pan C., Li M., Zhao X., Yu F., Zhao X., Lu G. (2022). Phytochrome interacting factor 3 regulates pollen mitotic division through auxin signalling and sugar metabolism pathways in tomato. New Phytol..

